# Drug Therapy Trials for the Prevention of Bronchopulmonary Dysplasia: Current and Future Targets

**DOI:** 10.3389/fped.2014.00076

**Published:** 2014-07-25

**Authors:** Vineet Bhandari

**Affiliations:** ^1^Division of Perinatal Medicine, Department of Pediatrics, Yale University School of Medicine, New Haven, CT, USA

**Keywords:** newborn, clinical trials, drug therapy, lung, chronic lung disease

Bronchopulmonary dysplasia (BPD), currently the most common chronic respiratory disease in infants, is a multifactorial disease secondary to genetic (1) and environmental factors (chief among them being exposure to invasive mechanical ventilation, ante- and postnatal infections, and hyperoxia) (2, 3). It is estimated that approximately 10,000–15,000 new cases of BPD occur each year in the United States, of which 97% occur in infants with birth weights <1250 g (3). Over the last decade, the incidence of BPD has been reported variably to have decreased (4), remained the same (5), or even increased slightly (3, 6, 7). However, there is uniform agreement that BPD is associated with significant resource utilization and increased costs (4, 8). While studies assessing the economic burden of BPD are mostly restricted to their initial hospitalization in neonatal intensive care units (4, 8), this is a chronic lung disease with significant pulmonary and neurodevelopmental sequelae (3, 9, 10) that impacts healthcare costs into the pediatric age group (11, 12) and would be expected to continue to do so into adulthood (13).

Given the above data, novel effective drug therapies for the prevention of BPD would potentially make a significant difference in the health and costs for prematurely born children. A recent workshop conducted under the auspices of the National Heart Lung and Blood Institute of the National Institutes of Health on the primary prevention of chronic lung diseases focused on BPD (14). In terms of “promising near-term opportunities for primary BPD prevention research,” specifically, “clinical research priorities and specific clinical trials for BPD prevention,” it was disappointing to note that only two specific drugs were named: caffeine and inhaled nitric oxide (iNO).

While caffeine has been associated with improvement in BPD (15) and neurodevelopmental outcomes (16) [unfortunately, not sustained at 5 years of age (17)], studies fine-tuning the timing of initiation and duration of use of this drug would be useful. This is important given the fact that the mechanism of action in terms of the pulmonary effects in the developing lung is not currently understood and toxicity concerns have been raised in an animal study (18). Despite a large number of infants being studied in randomized clinical trials (RCTs), iNO has not been consistently found to be beneficial in preventing BPD and is currently not recommended for such treatment (19, 20). It is therefore critical that for both caffeine and iNO, given past experience, sub-group targeted therapy (21) should be tested in future RCTs. Such targeted sub-groups could be on the basis of genotype or phenotype (for e.g., small for gestational age infants) criteria. Assessment of genotypes would be a useful technique to identify specific populations most likely to benefit from such a targeted approach, which would incorporate the not insignificant effects of the genetic contribution to BPD (1, 22–24).

On searching the *clinicaltrials.gov* database with the terms “drugs” and “BPD” (accessed on May 15, 2014; including only “open” studies that are actively recruiting; excluding those with “unknown status”), 24 studies were identified. Among these, those with specific drug therapy with the primary or secondary outcome listed as assessment of BPD included caffeine (1 trial), recombinant human Clara Cell 10 kDa protein (1 trial), iNO (2 trials), macrolide antibiotics (2 trials), hydrocortisone (2 trials), vitamin D (1 trial), remifentanil (1 trial), appropriate levels of oxygen (1 trial), maternal *N*-acetyl cysteine (1 trial), maternal enoxaparin (1 trial), and l-thyroxine (1 trial).

While awaiting the results of these clinical trials over the next few years, it is important that currently used drugs (approved for use in non-BPD medical conditions) be also tested and new drugs be developed to target novel molecular targets that have been identified in studies conducted in animal models of BPD (25). This becomes especially important since the incidence of BPD appears to be the same or slightly increased (25), despite the continuing use of non-invasive ventilation strategies (26). For translational impact, molecular targets that have been identified to be associated with human BPD would have the maximal potential to be of clinical use. Such potential therapies include anti-interleukin-1 (anakinra) (27), inhibition of Cox-2 and C/EBP homologous protein (CHOP) (celecoxib) (28, 29), targeting transforming growth factor-beta 1 signaling (losartan) (30), matrix proteins [elastase (elafin) (31), matrix metalloproteinase-9 (doxycycline) (28)], augmentation of the parathyroid hormone-related protein-peroxisome proliferator-activated receptor-gamma pathway (rosiglitazone, pioglitazone) (32, 33), modulation of macrophage migration inhibiting factor (34–36), and chitinase-3-like protein 1 (37, 38). Appropriate protocols for testing such drugs and/or their safer analogs in the preterm newborn population would need to be developed and it is imperative that data from studies conducted in older children and adults not be interpolated to the neonatal subjects, but independently verified. Strategies that incorporate drug delivery confined to the pulmonary compartment would minimize off-target effects (including untoward effects) and maximize the therapeutic response. For this to occur, given the current fiscal climate of federal funding, it is imperative that private philanthropic foundations with an interest in improving the health of children as well as pharmaceutical companies step up to the plate to partner with innovative physician-scientists to support pre-clinical/phase-1 studies of such drug therapies.

## Conflict of Interest Statement

The author declares that the research was conducted in the absence of any commercial or financial relationships that could be construed as a potential conflict of interest.
